# Aversive medical treatments signal a need for support: a mathematical model

**DOI:** 10.1017/ehs.2019.2

**Published:** 2019-05-28

**Authors:** Mícheál de Barra, Daniel Cownden, Fredrik Jansson

**Affiliations:** 1 Centre for Culture and Evolution, Brunel University, London; 2 Unaffiliated; 3 Centre for Cultural Evolution, Stockholm University; 4 Division of Applied Mathematics, Mälardalen University

**Keywords:** Cultural evolution, medical anthropology, sick role, iatrogenic disease, evolutionary medicine, cooperation, secondary gain

## Abstract

Ineffective, aversive and harmful medical treatments are common cross-culturally, historically and today. Using evolutionary game theory, we develop the following model to explain their persistence. Humans are often incapacitated by illness and injury, and are unusually dependent on care from others during convalescence. However, such caregiving is vulnerable to exploitation via illness deception, whereby people feign or exaggerate illness in order to gain access to care. Our model demonstrates that aversive treatments can counter-intuitively increase the range of conditions where caregiving is evolutionarily viable, because only individuals who stand to gain substantially from care will accept the treatment. Thus, contemporary and historical “ineffective” treatments may be solutions to the problem of allocating care to people whose true need is difficult to discern.

## Graphical abstract

**Figure:**
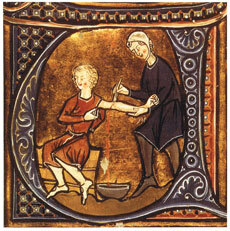

**Media summary:** Harmful medicine's ability to provide trustable evidence of patients’ need for care may explain its puzzling persistence.

## 1. Introduction

The idea that medicine should be unpleasant and aversive is well rooted in the English language. To *take one's medicine* is synonymous with enduring a deserved painful or unpleasant experience. Cheats who are themselves cheated *get a taste of their own medicine*. This reputation is well earned: historical medical treatments were often repugnant, dangerous, taboo breaking or painful. Widespread procedures included ingestion of substances like animal wastes, bird nests and human flesh, as well as poisons, emetics and diuretics. Surgical procedures like bloodletting, cupping and the reopening of partially healed wounds were common, as was forced feeding or food and water restrictions (Edgerton 1992; Miton and Mercier 2015; Sugg 2008; Wootton 2006). Within the history of medicine, the idea that a substantial proportion of pre-twentieth-century Western medicines were ineffective or harmful is uncontroversial (Hardy 2006; Wootton 2006).

The long-term popularity of harmful medicine is surprising given that, all else equal, one might expect individual and social learning processes to be biased against adopting cultural innovations that make life poorer, shorter or more difficult (Boyd *et al.* 2013). It is also puzzling that these medical treatments should be so *unpleasant and aversive*. Patients who opted for a warm bath over bloodletting sacrificed no therapeutic value. Yet rather than evolving towards gentle, pleasant or comforting treatments, the medicine that persisted was invasive, macabre and painful – often theatrically so.

One medical intervention, however, is ancient, common and unambiguously beneficial: caregiving. Caregiving involves keeping patients comfortable, providing food and other resources, and may also entail releasing people from duties and providing for their dependents. Cross-cultural research indicates that this kind of care is both essential and widespread. Some anthropologists have argued that human life history is premised on access to caregiving (Kaplan *et al.* 2000; Sugiyama 2004). In small-scale societies, people are often incapacitated by illness or injury and spend protracted periods of time unable to provide for themselves (Hill and Hurtado 2009). For example, Sugiyama (2004) reports that 90% of Shiwiar – forager-horticulturists in the Ecuadorian Amazon – had spent a fortnight or longer incapacitated. Sixty per cent of people fared even worse, spending a month or more unable to forage for themselves or their dependants. Without caregiving, an illness or injury of this duration is fatal. However, when people are disabled by disease, others provide food and other care and take over gardening tasks, sometimes for long periods. In North America about one in seven people provide care to a friend or family member who is ill or disabled in a given year (Marks 1996). A proportion of 2.5% of working hours in the UK are lost to sick leave – institutionalised caregiving – and more than half of these are due to minor illnesses or musculoskeletal illnesses (Comer 2017).

Caregiving is costly to the carer. Sugiyama (2004) tells how “two informants reported that they jointly maintained [a sick woman's] gardens for three months, but stopped when they could no longer sustain the work”. In contemporary Western societies, people involved in long-term caregiving experience poorer health (Vitaliano *et al.* 2003) and increased mortality risk (Perkins *et al*. 2012; Schulz and Beach 1999), suggesting that caregiving costs remain important even when health insurance and/or public health care provision exist.

From an evolutionary perspective, these costs often constitute a wise investment: helping a sick relative through a period of incapacity can have a substantial effect on their and their offspring's mortality. Hamilton's criterion (Hamilton 1964) for the evolution of care is frequently met (*c* < *rb*, where *r* is relatedness, *b* is benefit to the sick, and *c* is cost to carer). This accords with the cross-cultural and historical research discussed above – caregiving is common and important.

### 1.1 Illness deception

Caregiving, however, is open to exploitation via illness deception. From an evolutionary perspective, the problem is simple: the range of conditions where recipients should request care (*rc* < *b*) is much broader than the range of conditions where donors should be willing to grant care (*c* < *rb*) (Trivers 1974 highlights a similar conflict in the context of parental care). If illness were *transparent* – that is, donors could accurately estimate how much the recipient would benefit – then this would be of little consequence. Care could be granted only when it benefited inclusive fitness. However, health status is usually *opaque*. Many debilitating illnesses leave little visible sign upon the body, for example, back pain, hernia, kidney stones, gallstones, diabetes, Lyme disease and brucellosis. Conversely, many people with visible aberrations (scarring, rashes, disfigurement) are not in any need of care. Even among people with clear illnesses, it is difficult to estimate how much they will benefit from a given transfer of resources. There is good evidence that people harness this ambiguity in order to access caregiving which the donor would not be willing to offer had they complete information about the recipient's disease state.

Hysteria, malingering, factitious disorder, secondary gain and somatisation disorder are terms used to describe a cluster of related phenomena whereby people assume an ill social state without having a commensurate underlying pathology. They differ in the degree to which they seek release from a specific duty vs the general emotional and practical benefits of caregiving, and in the degree to which the deception is consciously planned and executed vs subconsciously motivated or reinforced. Here we refer to any attempt to feign or exaggerate illness which may result in access to caregiving as *illness deception*, irrespective of whether the behaviour is unconsciously or consciously motivated, and irrespective of whether the scale of the deception is severe or more trivial.

Illness deception is common. In one survey of clinical neuropsychologists, 30% of personal injury cases and 33% of disability and worker's compensation cases were judged to “probably” involve malingering or symptom exaggeration (Mittenberg *et al.* 2002). Several authors have highlighted how fluctuations in illness compensation claims appear unrelated to disease prevalence (Gun 1990; Nicholson and Martelli 2007). The introduction of compensation processes is associated with increasing pain reports and reduced treatment effectiveness (Rohling *et al.* 1995), and studies have demonstrated that actors can fool health professionals reasonably easily (Norman *et al.* 1982). Illness deception has also been documented in the historical record (see e.g. Withey 2013). As several authors have argued (Fabrega 1997; Finlay and Syal 2014; Steinkopf 2015, 2016; Tiokhin 2016), the fitness benefits associated with care may have acted as a selection pressure on symptom presentation. However, such a selection pressure may not always result in honest displays.

### 1.2 Aversive medicine maintains honesty

Caregiving can enhance the inclusive fitness of both donor and recipient, but it is vulnerable to exploitation via illness deception. We propose that decreasing the benefit of caregiving via aversive medical treatments can increase the range of conditions where caregiving is evolutionarily viable. This counter-intuitive proposal can be understood as follows: a fixed reduction in the benefit of care via aversive treatment can shift conditions so that illness deception is no longer viable, allowing caregiving to increase in frequency. These added costs to receiving treatment keep communication honest, by allowing caregivers to avoid the problem of distinguishing the ill from the illness deceivers, and by allowing those with hard-to-detect or easily imitated illnesses to credibly request care.

This result is possible because truly sick people have much more to gain from caregiving than someone engaged in illness deception. For someone with a significant illness, caregiving can prevent death. For someone with a minor illness or no disease, caregiving provides a lesser benefit, like release from duty or additional food. From an evolutionary perspective, if the aversive treatment (e.g. bloodletting or emetics) is of the appropriate cost, then illness deception will not benefit the individual. Concluding his review of symptoms-as-signals, Tiokhin (2016) independently arrives at a similar suggestion, noting that if “harsh treatments are painful and time consuming, the costs of treatment may not be worth it for those feigning injury”.

To better understand the circumstances where aversive treatments can enable caregiving to persist, we develop a mathematical model. Models help to direct our attention to key assumptions, as well as suggest predictions that might be tested in the future.

## 2. Model

We formulate an evolutionary model where individuals reproduce asexually, can be healthy or sick, and where they meet other individuals in random interactions. In these interactions, people have a strategy of whether to ask for help, at a cost to the helper (that causes reduction in fecundity) and a benefit (increasing fecundity) to the recipient, if provided, and whether to provide help when asked. Interactions are assorted, such that relatives meet at a certain frequency.

We use evolutionary game theory with fundamental ideas from invasion analysis (Maynard Smith and Price 1973) to explore the interaction of illness deception, harmful medicine, and caregiving. Specifically, we are interested in the conditions under which providing help is a stable strategy and those where it is not. Our main question is whether the range of conditions where helping is evolutionarily viable can be increased through the introduction of aversive medicine. We will first specify the evolutionary model, then describe the simplifying assumptions, and finally derive conditions for helping and asking strategies to be maintained in the population. For clarity, we keep the model simple, with a minimal set of possible strategies, illustrating the main idea of why aversive treatment can be adaptive, and persist even when only benign caregiving cannot. In the Supplementary Material, we expand upon this model with more strategies for what kind of treatment to provide and accept.

### 2.1 Specification and assumptions

An individual encounters the *opportunity* to make fraudulent requests for care (ask for help when healthy) with frequency *f*_h_, and the opportunity to make honest requests for care (ask for help when sick) with frequency *f*_s_ (whether an individual will actually make or receive a fraudulent or honest request depends on the strategy of the requester). We assume that these frequencies are set at the population level, that is, they are the same for all individuals. Since every opportunity for an individual to request care when ill is paired with an opportunity for another individual to provide that help (conditional on the request being made), an individual encounters the potential opportunities to provide help with the same frequencies: *f*_s_ to a sick individual and *f*_h_ to a healthy individual. When asked for help, an individual does not know whether the requester is sick or healthy.

Providing care entails a cost *c*. Receiving help gives a benefit *b*_s_ if the recipient is sick, and *b*_h_ if she is healthy. We assume throughout that the benefit of care when sick is greater than that when healthy, *b*_s_ > *b*_h_.

Finally, we assume that there is an assortative mechanism that produces a degree of relatedness *r* between interacting individuals. Relatedness is here defined as the probability that an allele sampled from the actor will be identical by descent to an allele sampled from the recipient, and hence they will employ the same strategy. We return to this assumption below. The variables of the model are summarised in Table 1.

**Table 1.** The variables of the model

Variable	Description
*f*_s_	Frequency of opportunity to ask for/give care where recipient is sick
*f*_h_	Frequency of opportunity to ask for/give care where recipient is healthy
*b*_s_	Benefit of care to sick
*b*_h_	Benefit of care to healthy
*c*	Cost of giving care
*r*	Degree of relatedness

To derive conditions for *helping* to be maintained in this model, the general idea is to consider the situations where there is a resident strategy at dynamic equilibrium, and evaluate the initial growth rate of a mutant strategy in such an environment, the invasion fitness (see e.g. Brännström *et al.* 2013). The success of the mutant strategy is then inferred by the growth rate when rare. As is common in invasion analysis, we incorporate the simplifying assumptions that the strategies interact within an infinite monomorphic population, that reproduction is asexual and that interaction occurs between pairs of strategies. Although these behaviours are cultural traits, our model focuses on the genetic fitness of people who engage in these behaviours. Later, we discuss how this genetic fitness might translate into cultural success.

Returning to the degree of relatedness *r*, suppose that there is a behaviourally relevant allele that causes reduction in personal fecundity *c* (for cost) while at the same time causing the fecundity of some other individuals to be increased by *b* (for benefit). Hamilton (1963) showed that in the case of discrete, non-overlapping generations, this allele for a helping behaviour can spread provided that there is some assortative mechanism whereby individuals are more likely to interact with relatives. Specifically, helping behaviour will be favoured by natural selection precisely when *rb* > *c*. While this seems relatively straightforward, it should be noted that many of the plausible assortative mechanisms that might cause interactants to be related, for example spatial structure coupled with limited offspring dispersal, can also serve to localise competition so that the benefits of cooperation are squandered in subsequent competition between relatives (West *et al.* 2002). Here we assume that competition is homogeneous throughout the population, such that competition is not stronger among relatives than among non-kin. This is a simplification, but since our aim is to illustrate the evolutionary potential of aversive medicine rather than to derive exact conditions for values of parameters (that lack empirical data), simplicity and clarity are more important.

Relatedness *r* is thus an input parameter to the model, and is the same throughout the population (as in the signalling model by Maynard Smith 1991; this is a first-order approximation for frequency change, or a “weak selection” assumption, as described by Rousset and Billiard 2000). In a scenario with several strategies in the population, this could potentially have a large impact on the dynamics, if we can expect that the success of different strategies will influence *r*. In our analysis, however, we compare the fitness of residents with the same strategy only to mutants with another strategy, similar to the approach taken by Taylor and Frank (1996), where *r* remains the same for a rare mutant (see also Gardner and West 2006, on the relative merits of approaches with closed models where *r* is determined by demographic assumptions vs open models where it is allowed to vary independently). In fact, as will be obvious in the invasion analysis, *r* is only relevant in the fitness equation for the mutant, so in our analysis, *r* can be interpreted as the frequency with which mutants interact with individuals identical by descent, and 1 − *r* as the frequency with which they interact with the rest of the population.

As mentioned earlier, while the person requesting care knows whether they are healthy or sick, the person receiving the request does not (health status is opaque). The possible strategies in this game are thus composed of three components: (1) whether or not to request care when ill; (2) whether or not to request care when healthy; and (3) whether or not to provide care when it is requested. This means that there are eight (2^3^) possible strategies, allowing for all possible combinations of the component parts of the strategies. However, three of these strategies weakly dominate the rest and so we limit our analysis to these three. The three dominant strategies are *Deceptive Nonhelper*, which will request care both when ill and when healthy and does not provide care when asked, *Honest Helper*, which requests care only when truly ill and provides care when asked, and *Deceptive Helper*, which requests care both when ill and healthy and provides care when asked. Since there is only one non-helping strategy, and no honest non-helpers, we will henceforth refer to *Deceptive Nonhelper* simply as *Nonhelper*. (Weak domination means that any strategy outside of the set of *Nonhelper*, *Honest Helper* and *Deceptive Helper* can only ever do as well as, but never better than, one of these dominating strategies, regardless of the population profile. All three dominant strategies ask for help when sick. The strategies that are dominated are Honest Nonhelper, and the corresponding strategies to the Honest Nonhelper and the three dominant ones that do not ask for help when sick.)

Let *δ* be the indicator function, that is, *δ*(*x*) = 1 if *x* is true and *δ*(*x*) = 0 if *x* is false. The general equation for any of these strategies is
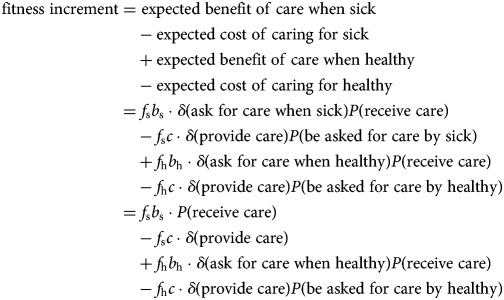


In each of these expressions, fitness is computed as the expected benefit or cost in the following four situations: asking for help as a sick person (the opportunity of which occurs with frequency *f*_s_ and provides a benefit *b*_s_ with probability *P*(receive care) since all strategies ask when sick); being asked for help by a sick person (with frequency *f*_s_, providing a cost *c* if the strategy provides care); having the opportunity to ask for help as a healthy person (with frequency *f*_h_, providing a benefit *b*_h_ with probability *P*(receive care) if the strategy asks for help when healthy); and potentially being asked for help by a healthy person (with frequency *f*_h_, providing a cost *c* if the strategy provides care and the recipient asks for it when healthy). We examine the fitness expressions for each strategy in turn.

We let *P*_N_, *P*_H_ and *P*_D_ denote the proportions in the population of *(Deceptive) Nonhelper*, *Honest Helper* and *Deceptive Helper* strategies, respectively, and let *W*_N_, *W*_H_ and *W*_D_ denote their respective fitness. Then we can compute the fitness benefit for *Nonhelpers* as
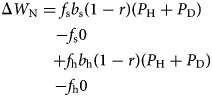


A *Nonhelper* always asks for help, but will receive it only when asking a non-relative (since relatives employ the same strategy, and thus never help), which occurs with probability 1 − *r*, who employs one of the helping strategies, which occurs with probability (*P*_H_ + *P*_D_). A *Nonhelper* never provides care.

The fitness benefit for *Honest Helpers* is
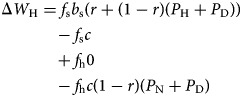


An *Honest Helper* will be helped when sick also by a relative, increasing the probability of receiving care when sick to *r* + (1 − *r*)(*P*_H_ + *P*_D_), while she never asks for help when healthy. An *Honest Helper* provides care when asked (and will always be asked if the recipient is sick). If the recipient is healthy, only a non-relative who employs one of the always asking strategies will use the opportunity to ask for help, which occurs with probability (1 − *r*)(*P*_N_ + *P*_D_).

Finally, for *Deceptive Helpers*, we have
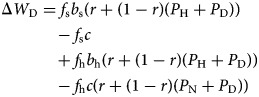


A *Deceptive Helper* is not different from an *Honest Helper* when sick. Given the opportunity, a *Deceptive Helper* will ask for help also when healthy, and has the same probability to receive it as when sick. Contrasting with *Honest Helper*, a *Deceptive Helper* will be asked for help by a healthy relative, increasing the probability of providing care for healthy to *r* + (1 − *r*)(*P*_N_ + *P*_D_).

### 2.2 Evolutionarily stable strategies

Using these expressions for fitness, we can investigate the conditions under which each pure strategy is resistant to invasion from rare mutants of the other strategies, that is, the *evolutionarily stable strategies* (ESS) (Maynard Smith and Price 1973). The invasion conditions are derived in the Supplementary Materials, and are summarised in Table 2.

**Table 2.** Conditions for when mutant can invade single resident strategy

Mutant\resident	Nonhelper	Honest helper	Deceptive helper
Nonhelper		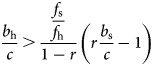	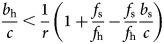
Honest helper	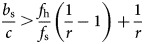		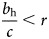
Deceptive helper	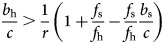	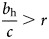	

The outcomes of these conditions can be visualised as a map in a parameter space. In the plots that follow, relatedness has been fixed at *r* = 0.25 and the frequencies have been fixed at *f*_s_ = *f*_h_ = 0.25, and we plot using the normalised parameters *b*_h_/*c* and *b*_s_/*c*, since it is the ratio of cost-to-benefit that determines the evolutionary outcomes, not the absolute values (see also Table 2). In the Supplementary Material, we show that qualitatively similar results hold for a range of *r*, *f*_s_ and *f*_h_ values.

In one of the limiting cases, when *c* < *rb*_h_ (which implies that *c* < *rb*_s_ since we assume *b*_h_ < *b*_s_), we have from Hamilton's rule that *Deceptive Helper* is the only ESS; in the other extreme case where *c* > *rb*_s_ (which again implies that *c* > *rb*_h_), Hamilton's rule gives *(Deceptive) Nonhelper* as the only ESS. The interesting cases are thus in the parameter range where *rb*_h_ < *c* < *rb*_s_, that is, where helping the ill is evolutionarily viable, but helping the healthy is not.

**Figure 1.**
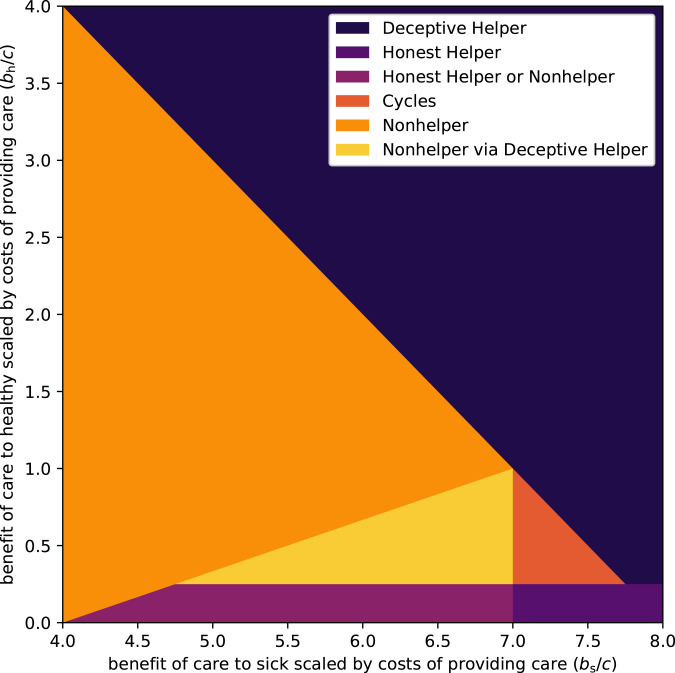
Evolutionarily stable strategies when relative benefit to sick (*b*_s_/*c*) and relative benefit to healthy (*b*_h_/*c*) vary. Relatedness is set to *r* = 0.25, and opportunities for illness deception and legitimate care request occur with equal probability, *f*_s_ = *f*_h_ = 0.25. Colours depict which pure strategies are stable for a given pair of benefits, for which they can resist invasion. The dark blue/purple regions are where helping strategies can be maintained: in the blue top-right region *Deceptive Helper* dominates; in the purple bottom-right region *Honest Helper* dominates; and in the violet bottom-left region *Honest Helper* and *Nonhelper* are in a stalemate situation where both are evolutionarily stable, with neither being able to invade the other. Helping is not maintained in the bright yellow/orange regions: in the orange left-most region *Nonhelper* dominates; in the yellow central-left triangular region, the dominance of *Nonhelper* is a direct result of the *Deceptive Helper* strategy being able to invade the *Honest Helper* strategy, paving the way for an invasion by Nonhelpers; and in the red central-right triangular region no strategy dominates, with *Honest Helper* being able to invade *Nonhelper*, which in turn is able to invade *Deceptive Helper*, which is in turn able to invade *Honest Helper*, and so on in a cycle.

**Figure 2.**
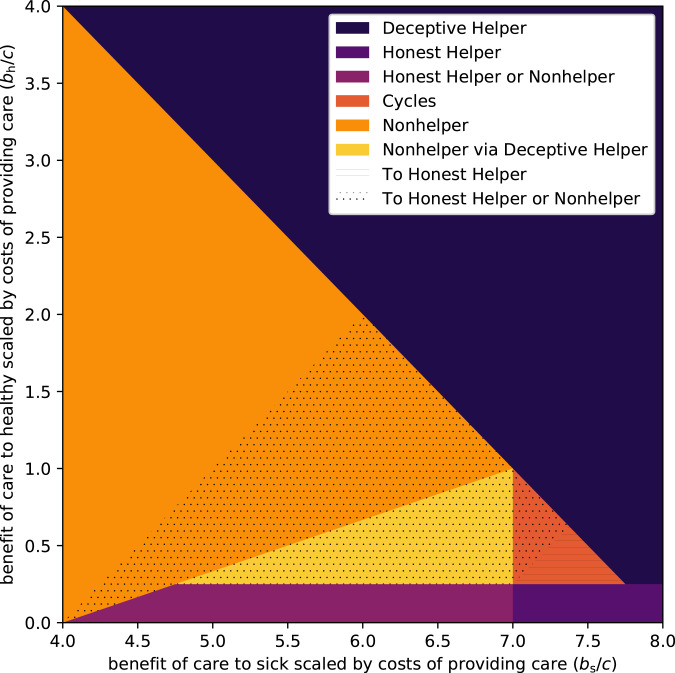
Evolutionarily stable strategies (ESSs) and potential helper ESSs when a harmful medical treatment is introduced. Relatedness is set to *r* = 0.25, and opportunities for illness deception and legitimate care request occur with equal probability, *f*_*s*_ = *f*_h_ = 0.25. The dotted region is where the dominance of *Nonhelper* (orange and yellow areas) can be eroded or cycling between *Nonhelper* and the other strategies (red area) can be stopped by aversive medicine, creating a stalemate situation where both *Nonhelper* and *Honest Helper* are evolutionarily stable. The lined area shows where aversive treatments can stop the cycling of strategies and make *Honest Helper* the sole ESS.

As can be seen in Figure 1, there is a broad range of conditions where the possibility of deception undermines caregiving (i.e. where *Nonhelpers* can establish – the light regions, mainly the orange and yellow regions, where they cannot be invaded, and to a lesser extent the dark orange region, where all strategies can be invaded). Now we consider the potential impact of aversive treatments on these evolutionary outcomes. For simplicity we assume that an aversive treatment reduces the benefit of receiving care when healthy and when sick in equal measure. Under this assumption, and in the context of Figures 1 and 2, the introduction of an aversive treatment can be thought of as shifting a model's point in the parameter space downward and to the left at a 45 degree angle (i.e. to follow a straight line with slope 1, in the left direction, where the length of the shift is determined by the aversiveness of the treatment). Figure 2 highlights those regions in the original parameter space where caregiving is undermined by illness deception (i.e. the light yellow/orange/red regions, where *Nonhelpers* can establish), but where it is possible for caregiving to become an ESS, via a judicious choice of the degree of aversiveness of the treatment. Figure A.1 in the Supplementary Material shows that, to the extent that illness deception undermines caregiving, aversive medicine can help prevent this erosion. That is to say, aversive medicine plays a more important role when illness deception is common.

In this model, we compare a universe where caregiving is benign and has no side effects with one where it is aversive, showing that treatment can become common where in the former universe it would not. However, the model does not allow for alternative practices to compete directly, and for caregiving and accepting treatment to be contingent on accepting aversive treatment when treatment without side effects may be a viable option. In the Supplementary Material, we extend the model to see whether aversive treatment can be sustained also in direct competition from benign treatment without side effects. Such a model significantly expands the number of possible strategies and makes the model less perspicuous, but, considering the same parameter space as in Figures 1 and 2, the results can be summarised as: (1) there will only be benign treatment where Deceptive Helpers constituted an ESS, but (2) the parameter space in which caregiving becomes possible owing to aversive treatment (the dotted and dashed regions) expands.

The following explicit empirical predictions are based on the original model, but the general qualitative predictions are consistent also with the extended model.

## 3. Empirical predictions

Here we outline how the theory and model generate predictions both about *when* we would expect to see harmful medicines and *how harmful* we would expect them to be. We also discuss how existing findings relate to these predictions and speculate on how they might be tested in the future.

### 3.1 No care without aversive treatment

If a function of medical treatment is to legitimise one's request for care, then people should be less inclined to provide care to those who do not undergo treatment, since if potential illness deceivers could access care without aversive treatment, then treatment's capacity to stabilise caregiving would evaporate. Thus, one prediction from our theory is that care will often be conditional on the acceptance of a treatment.

This prediction is consistent with Parson's (1951) sociological analysis of the *sick role*. When someone occupies a sick role, they are released from their social obligations and not held morally responsible for the additional burden that this places on others, but, crucially, they must do everything possible to exit the sick role, including taking any medications or treatments recommended by medical professionals. The theory outlined here suggests that this obligation to undergo the trials of treatment helps to maintain the stability of the institution in the face of would-be deceivers. Indeed, Parsons and Fox (1952) note how negative aspects of interaction with the health care system “are the penalties which give impetus to the patient's desire to re-achieve wellness”.

Qualitative research suggests that some patients have noted that access to care seems to be a function of treatment acceptance. A study of chronic pain suffering (Kleinman 1988, quoted by Glenton 2003) reports that:

The surgeries have had one clearly positive effect, in Howie's view. They have created icons of his travail, scars that he can show people, that he can touch himself to assure himself that there is something “physically wrong” with his back. After each of his surgeries, he felt that his family, fellow police officers, and doctors became more sympathetic. As he contemplates yet another major surgical procedure, this latent social function of surgery is a large part of the decision making, since his overall judgement about the surgeries is that they have made things worse.

Data on social support provided to patients who are randomised to undergo invasive or non-invasive treatments in clinical trials would provide an interesting test: if patient's need for care is opaque, then we would predict greater social support for patients who undergo the invasive procedure.

### 3.2 How harmful should treatments be?

In order to prevent illness deception from undermining caregiving, conditions must be set such that *Nonhelper* cannot invade. Table 2 shows that aversive medicine could potentially prevent *Nonhelper* from invading *Honest Helper*, but not from invading *Deceptive Helper* (since reducing *b*_h_/*c* and *b*_s_/*c* would decrease the left-hand side and increase the right-hand side of the inequality). However, some amount of harm could prevent *Deceptive Helper* from invading *Honest Helper*. Thus, there are two regions where aversive medicine could maintain caregiving: it can stop *Nonhelpers* from invading directly if
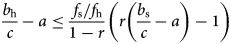
and from invading by way of *Deceptive Helper* if
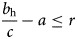
where *a* ≥ 0 is the amount of harm of an aversive treatment. The smallest amount of *a* that will meet the inequalities is thus
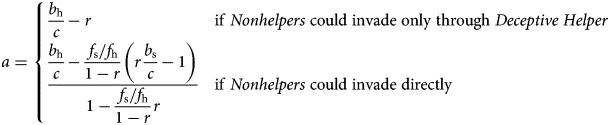


Note that the denominator in the second expression is negative if (and only if)
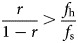
that is, when there are few opportunities to ask for help when healthy as compared with when sick, and/or most of these requests will be to relatives. For example, if *f*_s_ ≤ *f*_h_ and *r* < 0.5, then the denominator is positive.

The first expression increases with *b*_h_/*c*, and so does the second when the denominator is positive, so we would expect to see more severe treatments when the benefits of illness deception are great. Therefore we predict that treatments will be more harmful, for example, in times of intergroup conflict among potential combatants relative to times of peace or among non-potential combatants. Illness deception has long been a problem for armed forces. The problem became acute in the First World War owing to uncertainty over whether neuropsychological problems like “shell shock” were instances of illness deception. Wessely (2003) notes that the growing suspicion among military and medical elites, coupled with a shortage of men, meant that “German (and of course British) treatments for the war neuroses became increasingly punitive”.

A related prediction is that treatments should be more harmful in societies where people engage in dangerous foraging activities (e.g. hunting large mammals) than in societies where resource acquisition is safer: this hypothesis may be testable with cross-cultural ethnographic datasets. The idea that care is contingent on harsher treatment when the benefits of access to the sick role is higher might also be tested in vignette experiments.

On the other hand, to prevent *Deceptive Helper* from invading, we expect medicines to be less harmful when the denominator is large, that is, when the costs to the caregiver are substantial. This is somewhat counter-intuitive – would not a stronger deterrent be preferable when the costs of caregiving are large? However, it can be understood as a consequence of the fact that the cost of caregiving is disproportionately borne by relatives. Hence, from an inclusive fitness perspective, the costs to relatives of a request for care will not outweigh the benefits to self from that care. Finally, the closer the relatives are, the more benign an aversive treatment to stop *Deceptive Helpers* can be. We know of no data that test these predictions directly.

In the region where *Nonhelpers* can invade *Honest Helpers* directly, the aversiveness of medicine can either increase or decrease with costs and high relatedness, depending on the other variables. For example, if
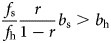
then medicines should be more harmful when the costs to the caregiver are large, while in the opposite case, they are expected to be less harmful. We refrain from going into further detail, the main point being that predictions are more complex when *Nonhelpers* can invade directly.

### 3.3 When should harmful medicine be more common?

Aversive medical treatments are expected to be more common when illness deception is possible. In situations where need is largely transparent – for example, when diagnostics are reliable, where the disease has obvious, familiar causes, or for an illness that is difficult to fake – then costly treatments are not needed. Epidemic infectious diseases that infect large numbers of people and that have consistent symptomatology and consequences will negate the need for harmful treatments. So will the reliable diagnostic and prognostic methods that have become common over recent decades.

The quotation above from the back pain sufferer (Kleinman 1988) illustrates the particular importance of visible and significant treatment when the visible symptoms are absent. Similarly, in her study of illness behaviour in Fiji, Trnka (2007) finds that women whose need for care is opaque seek costly legitimation of their problem via written doctors’ prescriptions, even though the medication they desire is widely available. We might predict that individuals with, for example, an obvious cut rather than non-obvious muscle injury would be less concerned about this prescription. A related prediction amenable to laboratory testing is that acceptance of costly treatment should be less relevant to would-be caregivers when the need is transparent.

Generally speaking, cultures that deploy aversive treatments for ailments where the costs of providing and the benefits of receiving care correspond to those in the hatched parameter space of Figure 2, are less likely to have their caregiving practices undermined by illness deception. This has a number of implications. When *c* is very low, caregivers have little to lose and much to gain by offering care freely. As *c* increases, we would expect costly treatments to become more common (until *c* becomes so substantial that the benefit to healthy scaled by costs to provider is close to zero, in which case costly treatments are again not needed); in Figure 2, this is equivalent to a move from the top right diagonally down and left into the hatched central area. In the real world a range of factors will influence *c*, including the caregiver's time or energy, food availability and the scale of the care requested.

It follows that childhood illnesses are less likely to be treated with harmful medicines. Even when healthy, children's economic contribution is limited and hence the loss of their labour is a less significant problem. Moreover, children require substantial care independent of illness. Thus *c* will generally be relatively low. For similar reasons, the elderly and infirm are also less likely to be treated with harmful medicines. Although the perceived absence of side-effects is an important reason why children are given complementary and alternative medicines (Cuzzolin *et al*. 2003), other datasets are needed to test this prediction more directly.

## 4. Discussion

Our model suggests that the judicious introduction of harmful treatments, in conjunction with effective caregiving, broadens the range of conditions where caregiving is evolutionarily viable. There is a broad range of conditions, that is, relative cost–benefit ratios of receiving and providing care, where the possibility of illness deception renders caregiving evolutionarily inviable. We show that the introduction of aversive medicine that reduces the benefit of receiving care can in some cases transform the underlying strategic situation so that caregiving becomes evolutionarily viable where previously it was not. This is possible because the benefit of care for the truly sick is greater than the benefit of care for the illness deceiver. The model shows that there is scope for the benefit of care to be reduced for both the ill and the illness deceivers in such a way that illness deception is no longer the evolutionarily dominant strategy, allowing caregiving the chance to increase in frequency.

Note that the current model has no bearing on the spread of *beneficial* or *effective* treatments. Treatment benefit and harm are orthogonal – a single treatment can be both very aversive and very helpful (e.g. surgery). We suggest that selection pressures sometimes favour treatments higher in the harm dimension, but it is plausible that other selection pressures may favour treatments higher in the benefit dimension, particularly because the benefit of effective treatments is often only realised if the recipient is truly ill. Some specific theoretical work as well as general cultural evolution models suggest that treatments may also evolve towards helpfulness (Henrich and Henrich 2010; Tanaka *et al.* 2009).

Although the model above analyses treatments as if they were genetic traits, medicine is largely a cultural phenomenon. However, there are several processes by which genetic fitness could translate into cultural fitness. Once a harmful medical practice emerges in a community, people who accept or demand the use of this signal will, on average, have better health than those who reject it. Better health translates into more, healthier, children, and thus if medical beliefs are passed from parent to child, its frequency will increase within the group. Moreover, people are probably more inclined to learn from healthy peers, parent and elders than from the ill. Thus oblique and horizontal transmission may also facilitate trait spread. Alternatively, if healthy individuals are better transmitters of cultural practices of medicine and helping behaviour, and transmission takes place in the same assorted interactions as the opportunities for help, then our model translates into a cultural evolution model, with fitness being a measure of cultural transmission from an individual. Another possibility is that as a result of individual learning, or cultural or genetic evolution, human cognition is generally sensitive to the risk of deception (including illness deception) as well as to cues (such as treatment acceptance) that indicate such deception is unlikely. Such a psychology would provide fertile ground for the cultural evolution of harmful therapies.

The value of harmful medicine is not dependent on people understanding its functional role. We suggest that over many generations, harmful medicine spreads within a community because people who use it end up healthier (and having healthier kin) than people who do not. “Deterrent” medicine may work better when its true function is hidden, since if the message component were obvious, then skilled illness deceivers might circumvent the treatment through persuasion or appeals to other kinds of evidence that purport to demonstrate their illness. Those who suspect illness deception would need to make an explicit accusation, an act likely to damage relationships, whether or not illness deception is taking place.

There are parallels between the processes described here and costly signalling theory (Grafen 1990; Zahavi 1975). However, in many costly signalling contexts, what varies is the costs of producing a given signal. In the present case, the cost of producing the signal is similar across all individuals. What varies is instead the benefit that results from the production of this signal such that people who are sicker stand to gain much more from a unit of care than people who are less sick. Hence the fixed cost of aversive treatments will deter all but people who stand to gain substantially. The chick begging model developed by Godfray (1991) has a similar dynamic. Chicks pay a cost to request food through begging, and the benefit of a unit of that food is lower if the chick has been recently fed. Like in the medical case outlined here, the fixed costs of requesting enable donors to efficiently identify situations where that transfer of resources is most useful. Unlike in the medical case, the transfer of resources is unidirectional, from parent to offspring.

While we have built this model within a kin selection framework, other processes may enable the evolution of caregiving as well as illness deception and harmful medical treatments. According to direct reciprocity theory (Trivers 1971), individuals will provide each other with care in times of need with the expectation that this care is reciprocated in the future. Care based on such reciprocity is less subject to erosion via illness deception, since the carer's fitness is enhanced by the return of care when they fall ill in the future. Thus, whether the care benefits the requester a lot (if they are truly sick) or a little (if they are engaging in deception) is of little consequence to the carer; they should only be concerned about the availability of care to themselves in the future. However, direct reciprocity depends on a predictability and symmetry of illness or injury that may be rare in nature, since people cannot predict if, when, and how much care they will need in the future (see also Clutton-Brock 2009; Raihani and Bshary 2011). Also, if they predict that the requester may never recover to a degree that would enable them to return the care, direct reciprocity alone will not sustain caregiving.

Indirect reciprocity (Nowak and Sigmund 1998), in which people with a *reputation as caring* are then cared for if they request it, may be more likely to sustain caregiving. Such a reputation-based system depends less on a symmetry of need between partners and the predictability of illness or injury. However, in a society where people are inclined to provide care so as to maintain a caring reputation, an incentive to engage in illness deception will exist. Since the amount of care available within this society is finite, frequent illness deception will diminish the care available to people with true illnesses. Thus, a sort of *tragedy of the commons* may result, whereby illness deception reduces the care available to the truly ill, who benefit much more from each unit of care. However, like in the kin selection model we developed here, harmful treatments that impose a fixed cost on every requester will diminish this problem, since only people who stand to benefit substantially from care will request it in the face of these costs. Moreover, aversive treatments may enable ill actors who require care to maintain an “honest” reputation; this may be important for maintaining or developing new cooperative relationships in contexts where partner selection occurs (Barclay 2013; Baumard *et al.* 2013).

In conclusion, the theory presented here suggests an explanation for several puzzling questions of medical cultural evolution and contributes to a growing literature on the evolution of medical practice (De Barra 2017; De Barra *et al.* 2014; Jiménez *et al.* 2018; Miton and Mercier 2015; Miton *et al.* 2015; Steinkopf 2017; Tanaka *et al*. 2009). Medicines may serve not just to cure disease but also to deter illness deception. Many treatments that are directly harmful may be indirectly beneficial in that they help to expand the range of circumstances in which valuable caregiving can persist.

## Supplementary material

For supplementary material accompanying this paper visit http://dx.doi.org/10.1017/ehs.2019.2. click here to view supplementary material
