# Supplementary material for: Aversive medical treatments signal a need for support: a mathematical model
Source: Evol Hum Sci. 2019 May 28;1:e4. doi: 10.1017/ehs.2019.2 (PMC10427312; doi:10.1017/ehs.2019.2)
Supplement: Supplementary file 1 [file S2513843X19000021sup001.zip › S2513843X19000021sup001/SuppMaterials.pdf]

Aversive medical treatments signal a need for support: a mathematical model

Mícheál de Barra, Daniel Cownden and Fredrik Jansson

A Supplementary material

A.1 Invasibility Conditions

Let f_s and f_h be the frequencies of opportunities to ask (or being asked) for help when sick and when healthy, respectively, let b_s and b_h be their associated benefits to the recipient if care is being provided, and c the cost to the provider. Further, assume that the frequency of interaction with kin is r. Let W_N, W_H and W_D denote the fitness for the three strategies *(Deceptive) Nonhelpers*, *Honest Helpers* and *Deceptive Helpers*, respectively, and let P_N, P_H and P_D denote their respective frequency in the population. The expressions for the change in fitness are then

$$
\begin{aligned}
\Delta W_\mathrm{N} &= f_\mathrm{s} b_\mathrm{s}(1-r)(P_\mathrm{H}+P_\mathrm{D}) \\
&\quad - f_\mathrm{s}0 \\
&\quad + f_\mathrm{h} b_\mathrm{h}(1-r)(P_\mathrm{H}+P_\mathrm{D}) \\
&\quad - f_\mathrm{h}0 \\
\Delta W_\mathrm{H} &= f_\mathrm{s} b_\mathrm{s}(r+(1-r)(P_\mathrm{H}+P_\mathrm{D})) \\
&\quad - f_\mathrm{s}c \\
&\quad + f_\mathrm{h}0 \\
&\quad - f_\mathrm{h}c(1-r)(P_\mathrm{N}+P_\mathrm{D}) \\
\Delta W_\mathrm{D} &= f_\mathrm{s} b_\mathrm{s}(r+(1-r)(P_\mathrm{H}+P_\mathrm{D})) \\
&\quad - f_\mathrm{s}c \\
&\quad + f_\mathrm{h} b_\mathrm{h}(r+(1-r)(P_\mathrm{H}+P_\mathrm{D})) \\
&\quad - f_\mathrm{h}c(r+(1-r)(P_\mathrm{N}+P_\mathrm{D}))
\end{aligned}
$$

In the main text we report the conditions under which a given strategy can resist invasions from the other strategies. What this means specifically is that the expected fitness of the resident strategy is greater than the expected fitness of the invading strategy when the population is composed, effectively exclusively, of the resident strategy.

We say that *Nonhelper* invades *Honest Helper* if in a population consisting almost entirely of *Honest Helper*, the *Nonhelper* strategy has higher fitness. So setting $P_{\mathrm{H}} = 1$ and $P_{\mathrm{D}} = P_{\mathrm{N}} = 0$, in the expressions for ΔW_{N} and ΔW_{H}, we obtain

$$\begin{aligned} & \Delta W_{\mathrm{N}} > \Delta W_{\mathrm{H}} \\ \iff \quad & (f_{\mathrm{s}} b_{\mathrm{s}} + f_{\mathrm{h}} b_{\mathrm{h}})(1-r) > f_{\mathrm{s}}(b_{\mathrm{s}} - c) \end{aligned}$$

That is, if the gains from exploiting the helpers when playing non-relatives experienced by rare mutant *Nonhelpers* are greater than the gains from efficient cooperation experienced by the population of *Honest Helpers*. To help visualise invasion conditions with regard to parameter values, we will rewrite each condition as the threshold value of b_{h}/c over which the invasion condition changes, for a given value of b_{s}/c. The condition for *Nonhelper* invading *Honest Helper* is thus rearranged as

$$\frac{b_{\mathrm{h}}}{c} > \frac{\frac{f_{\mathrm{s}}}{f_{\mathrm{h}}}}{1-r}\left(r\frac{b_{\mathrm{s}}}{c} - 1\right)$$

Setting $P_{\mathrm{D}} = 1$ and $P_{\mathrm{N}} = P_{\mathrm{H}} = 0$ we see that *Nonhelper* invades *Deceptive Helper* if

$$\begin{aligned} & \Delta W_{\mathrm{N}} > \Delta W_{\mathrm{D}} \\ \iff \quad & (f_{\mathrm{s}} b_{\mathrm{s}} + f_{\mathrm{h}} b_{\mathrm{h}})(1-r) > f_{\mathrm{s}}(b_{\mathrm{s}} - c) + f_{\mathrm{h}}(b_{\mathrm{h}} - c) \\ \iff \quad & r(f_{\mathrm{s}} b_{\mathrm{s}} + f_{\mathrm{h}} b_{\mathrm{h}}) < c(f_{\mathrm{s}} + f_{\mathrm{h}}) \end{aligned}$$

Nonhelper misses out on some benefit from not being helped by relatives, but they also avoid the costs of providing care. If, relative to the resident *Deceptive Helper* population, the benefits they miss out on, on the left-hand side, are less than the costs they avoid paying, on the right-hand side, then *Nonhelpers* can invade when rare. Rearranging this condition to isolate $\frac{b_{\mathrm{h}}}{c}$ yields

$$\frac{b_{\mathrm{h}}}{c} < \frac{1}{r}\left(1 + \frac{f_{\mathrm{s}}}{f_{\mathrm{h}}} - \frac{f_{\mathrm{s}}}{f_{\mathrm{h}}}\frac{b_{\mathrm{s}}}{c}\right)$$

Setting $P_{\mathrm{N}} = 1$ and $P_{\mathrm{H}} = P_{\mathrm{D}} = 0$, we see that *Honest Helper* invades resident *Nonhelper*

if

$$\begin{aligned} \Delta W_{\mathrm{H}} &> \Delta W_{\mathrm{N}} \\ \iff \quad f_{\mathrm{s}} b_{\mathrm{s}} r - f_{\mathrm{s}} c - f_{\mathrm{h}} c(1-r) &> 0 \\ \iff \quad f_{\mathrm{s}} b_{\mathrm{s}} r &> c(f_{\mathrm{s}} + f_{\mathrm{h}}(1-r)) \end{aligned}$$

That is, if the benefit of help from kin to *Honest Helper* is greater than the cost of providing help to both kin and the exploitative *Nonhelper* population. Note that this condition does not depend on b_{h}, only on b_{s}. Isolating $\frac{b_{\mathrm{s}}}{c}$, we have,

$$\frac{b_{\mathrm{s}}}{c} > \frac{f_{\mathrm{h}}}{f_{\mathrm{s}}}\left(\frac{1}{r} - 1\right) + \frac{1}{r}$$

Setting $P_{\mathrm{D}} = 1$ and $P_{\mathrm{H}} = P_{\mathrm{N}} = 0$, we see that *Honest Helper* invades resident *Deceptive Helper* if

$$\begin{aligned} \Delta W_{\mathrm{H}} &> \Delta W_{\mathrm{D}} \\ \iff \quad f_{\mathrm{s}} b_{\mathrm{s}} - f_{\mathrm{s}} c - f_{\mathrm{h}} c(1-r) &> f_{\mathrm{s}} b_{\mathrm{s}} - f_{\mathrm{s}} c + f_{\mathrm{h}} b_{\mathrm{h}} - f_{\mathrm{h}} c \\ \iff \quad -f_{\mathrm{h}} c(1-r) &> f_{\mathrm{h}} b_{\mathrm{h}} - f_{\mathrm{h}} c \\ \iff \quad cr &> b_{\mathrm{h}} \end{aligned}$$

Deceptive Helper bears the cost of helping healthy kin, but *Honest Helper* does not. If this cost is greater than the benefit of receiving help when healthy, then *Honest Helper* can invade. This condition does not depend on b_{s} and can be rewritten as

$$r > \frac{b_{\mathrm{h}}}{c}$$

Setting $P_{\mathrm{N}} = 1$ and $P_{\mathrm{H}} = P_{\mathrm{D}} = 0$, we see that *Deceptive Helper* invades resident *Nonhelper* if

$$\begin{aligned} \Delta W_{\mathrm{D}} &> \Delta W_{\mathrm{N}} \\ \iff \quad f_{\mathrm{s}} b_{\mathrm{s}} r - f_{\mathrm{s}} c + f_{\mathrm{h}} b_{\mathrm{h}} r - f_{\mathrm{h}} c &> 0 \\ \iff \quad r(f_{\mathrm{s}} b_{\mathrm{s}} + f_{\mathrm{h}} b_{\mathrm{h}}) &> c(f_{\mathrm{s}} + f_{\mathrm{h}}) \end{aligned}$$

So *Deceptive Helper* can invade when the benefit of help from kin is greater than the cost of providing help to both kin and the exploitative *Nonhelper* population. Note that this is just the reverse of the inequality for when *Nonhelper* can invade Deceptive Helper, which when rearranged to yield the critical value of $\frac{b_{\mathrm{h}}}{c}$ as a function of $\frac{b_{\mathrm{s}}}{c}$ gives

$$\frac{b_{\mathrm{h}}}{c} > \frac{1}{r}\left(1 + \frac{f_{\mathrm{s}}}{f_{\mathrm{h}}} - \frac{f_{\mathrm{s}}}{f_{\mathrm{h}}}\frac{b_{\mathrm{s}}}{c}\right)$$

Setting $P_{\mathrm{H}} = 1$ and $P_{\mathrm{D}} = P_{\mathrm{N}} = 0$, we see that *Deceptive Helper* invades resident *Honest*

Helper if

$$\begin{aligned} & \Delta W_{\mathrm{D}} > \Delta W_{\mathrm{H}} \\ \iff \quad & f_{\mathrm{s}}(b_{\mathrm{s}} - c) + f_{\mathrm{h}}(b_{\mathrm{h}} - cr) > f_{\mathrm{s}}(b_{\mathrm{s}} - c) \\ \iff \quad & cr < b_{\mathrm{h}} \end{aligned}$$

The cost of helping healthy kin, which Deceptive Helpers must bear, is less than the benefit of receiving help when healthy. Rearranging this condition in terms of $\frac{b_{\mathrm{h}}}{c}$, we have

$$\frac{b_{\mathrm{h}}}{c} > r$$

Note that this is just the reverse of the condition where *Honest Helper* invades resident *Deceptive Helper*.

A.2 Different Parameter Values

In the main text, we found that aversive treatment could maintain helping behaviour under a wide range of conditions where it would otherwise have eroded, for the parameter values $r = 0.25$ and $f_{\mathrm{s}} = f_{\mathrm{h}} = 0.25$. In Figure A.1, we show that qualitatively similar results hold for a range of r, f_{s} and f_{h} values. Note though that when relatedness is high, and the frequency of opportunities for illness deception (f_{h}) are relatively low compared to the frequency of opportunities for legitimate requests for care (f_{s}), then the range of cost-benefit ratios where caregiving is evolutionarily viable is quite broad, and the scope of illness deception to undermine helping is relatively limited. In such cases, because illness deception is not such a problem, there are fewer situations where the introduction of aversive treatment will alter the evolutionary outcomes.

A.3 An Extended Model

Our main model investigates under what parameter values caregiving evolves in the absence or presence of aversive treatments, showing that under certain settings, aversive treatment would be sustained where treatment without side effects would not. However, the model does not allow for alternative practices to compete directly, and for caregiving and accepting treatment to be contingent on accepting aversive treatment when treatment without side effects may be a viable option.

We here extend our original model to see whether aversive treatment can be sustained also in direct competition from treatment without side effects. Such a model significantly expands the number of possible strategies and makes the model less perspicuous, so the main

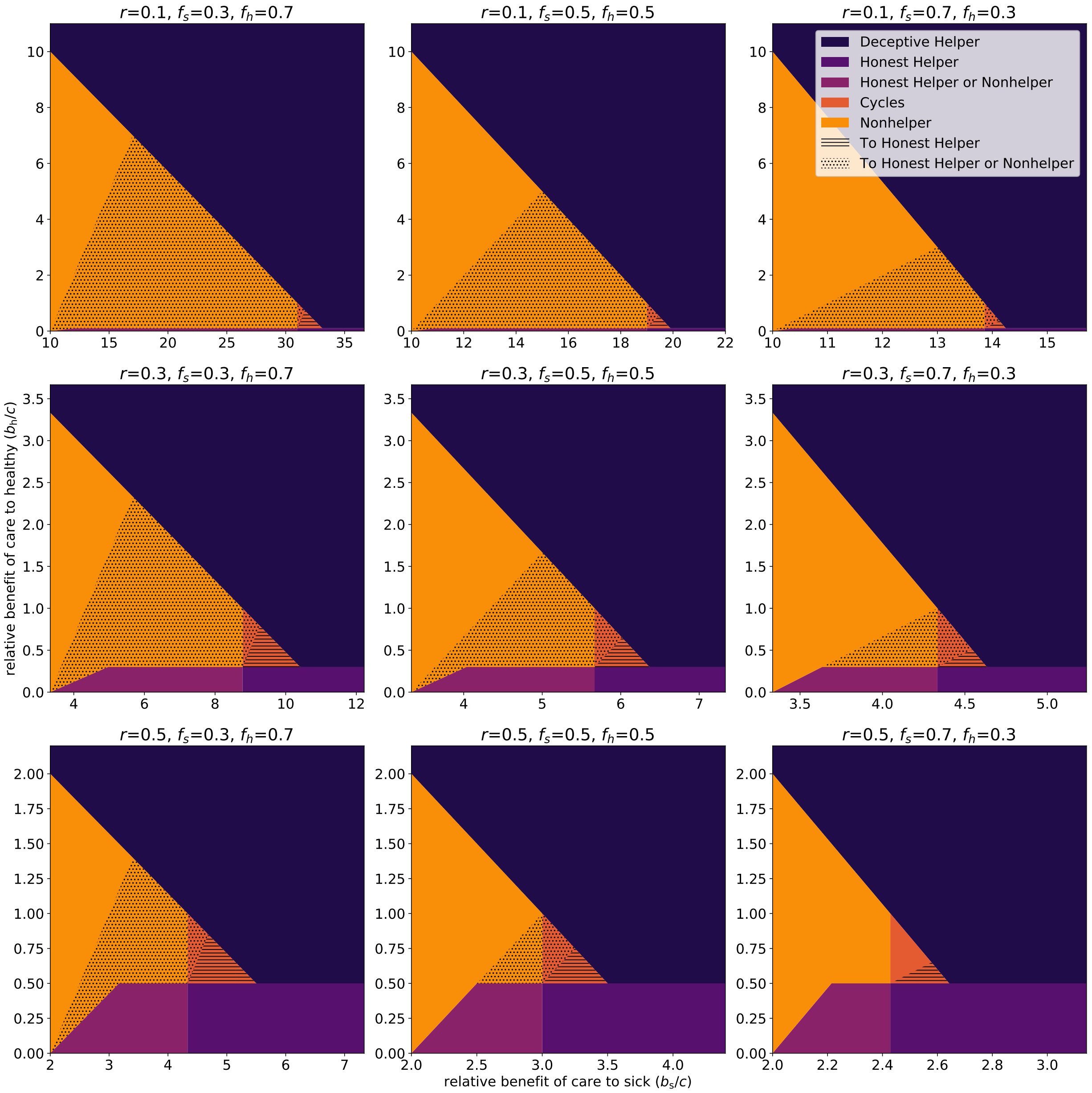

Figure A.1: Evolutionarily Stable Strategies when benefit to sick (b_{s}) and benefit to healthy (b_{h}) vary across different parameter ranges of $r = (0.1, 0.3, 0.5)$ from top to bottom, and $f_{\mathrm{s}} = (0.3, 0.5, 0.7)$ and $f_{\mathrm{h}} = (0.7, 0.5, 0.3)$ from left to right. Note that the scale of the axis in each of these subfigures is different.

aim of introducing it is for comparison to our original model, and to investigate whether aversive treatment can be maintained also when it is not the only option.

Instead of treatment vs. no treatment, there are now three choices: aversive treatment with costly effects for the receiver, reducing the benefit of receiving it; benign treatment without costs for the receiver; and no treatment. As before, caregiving (with aversive or benign treatment) imposes a cost on the donor.

This gives a total of $3^3 = 27$ possible strategies, consisting of:

- whether to provide no (N), benign (B) or aversive (A) treatment to those who ask for help,
- whether not to ask for help (N) or to ask for it and accept it only if it is benign (B) or to always accept it (A), when given the opportunity when you are sick,
- and the same consideration for when you are healthy.

Let W_{ijk} denote the fitness for an agent with strategy ijk, $i, j, k \in \{\mathrm{N}, \mathrm{F}, \mathrm{A}\}$, where i denotes the strategy when being asked for help, j the strategy when being sick and given the opportunity to ask for help, and k the strategy when being healthy and given the opportunity to ask for help.

There are five strategies that weakly dominate the remaining twenty-two (as can be realised for example by writing down all 27 fitness change equations), so our analysis will pertain to these. All five strategies entail asking for help when being sick and accepting all treatments, whether aversive or benign (we here assume that $b_h - a > c$, that is, that the net benefit to the sick receiver is positive, and greater than the cost to the donor). The strategies are:

- NAA (Deceptive nonhelper): Provide no help, but always ask for it and accept any kind of treatment.
- BAN (Honest benign helper): Provide help without side effects to anyone who asks for it, but do not ask for help when healthy.
- BAA (Deceptive benign helper): Provide help without side effects to anyone who asks for it, and always ask for help and accept any kind of treatment.
- AAB (Opportunistic aversive helper): Provide help with side effects to those who accept it, and always ask for help, but accept it when healthy only if it is benign.
- AAA (Deceptive aversive helper): Provide help with side effects to those who accept it, and always ask for help and accept any kind of treatment.

Note that two of the remaining five strategies do include aversive treatment. Note also that the strategies of the original model, (Deceptive) Nonhelper, Deceptive Helper and Honest Helper, are replicated for benign treatments, but in the aversive case, Honest Helper, AAN, is dominated by an opportunistic helper that accepts benign treatment when healthy.

Since all agents have the same strategy when sick, we will henceforth drop the middle index, and denote the fitness of each strategy by W_{ik} and the prevalence by P_{ik}, with i and k as above. Let a be the amount of reduction in benefit given by an aversive treatment. The changes in fitness after an interaction for agents with each of the five strategies are given by the following functions:

$$
\begin{aligned}
\Delta W_{\mathrm{NA}} &= f_{\mathrm{s}}(1-r)(b_{\mathrm{s}}(P_{\mathrm{BN}}+P_{\mathrm{BA}})+(b_{\mathrm{s}}-a)(P_{\mathrm{AB}}+P_{\mathrm{AA}})) \\
&\quad + f_{\mathrm{h}}(1-r)(b_{\mathrm{h}}(P_{\mathrm{BN}}+P_{\mathrm{BA}})+(b_{\mathrm{h}}-a)(P_{\mathrm{AB}}+P_{\mathrm{AA}})) \\
\Delta W_{\mathrm{BN}} &= f_{\mathrm{s}}(rb_{\mathrm{s}}+(1-r)(b_{\mathrm{s}}(P_{\mathrm{BN}}+P_{\mathrm{BA}})+(b_{\mathrm{s}}-a)(P_{\mathrm{AB}}+P_{\mathrm{AA}}))) \\
&\quad - f_{\mathrm{s}}c \\
&\quad - f_{\mathrm{h}}c(1-r)(1-P_{\mathrm{BN}}) \\
\Delta W_{\mathrm{BA}} &= f_{\mathrm{s}}(rb_{\mathrm{s}}+(1-r)(b_{\mathrm{s}}(P_{\mathrm{BN}}+P_{\mathrm{BA}})+(b_{\mathrm{s}}-a)(P_{\mathrm{AB}}+P_{\mathrm{AA}}))) \\
&\quad + f_{\mathrm{h}}(rb_{\mathrm{h}}+(1-r)(b_{\mathrm{h}}(P_{\mathrm{BN}}+P_{\mathrm{BA}})+(b_{\mathrm{h}}-a)(P_{\mathrm{AB}}+P_{\mathrm{AA}}))) \\
&\quad - f_{\mathrm{s}}c \\
&\quad - f_{\mathrm{h}}c(r+(1-r)(1-P_{\mathrm{BN}})) \\
\Delta W_{\mathrm{AB}} &= f_{\mathrm{s}}(r(b_{\mathrm{s}}-a)+(1-r)(b_{\mathrm{s}}(P_{\mathrm{BN}}+P_{\mathrm{BA}})+(b_{\mathrm{s}}-a)(P_{\mathrm{AB}}+P_{\mathrm{AA}}))) \\
&\quad + f_{\mathrm{h}}(1-r)b_{\mathrm{h}}(P_{\mathrm{BN}}+P_{\mathrm{BA}}) \\
&\quad - f_{\mathrm{s}}c \\
&\quad - f_{\mathrm{h}}c(1-r)(P_{\mathrm{NA}}+P_{\mathrm{BA}}+P_{\mathrm{AA}}) \\
\Delta W_{\mathrm{AA}} &= f_{\mathrm{s}}(r(b_{\mathrm{s}}-a)+(1-r)(b_{\mathrm{s}}(P_{\mathrm{BN}}+P_{\mathrm{BA}})+(b_{\mathrm{s}}-a)(P_{\mathrm{AB}}+P_{\mathrm{AA}}))) \\
&\quad + f_{\mathrm{h}}(r(b_{\mathrm{h}}-a)+(1-r)(b_{\mathrm{h}}(P_{\mathrm{BN}}+P_{\mathrm{BA}})+(b_{\mathrm{h}}-a)(P_{\mathrm{AB}}+P_{\mathrm{AA}}))) \\
&\quad - f_{\mathrm{s}}c \\
&\quad - f_{\mathrm{h}}c(r+(1-r)(P_{\mathrm{NA}}+P_{\mathrm{BA}}+P_{\mathrm{AA}}))
\end{aligned}
$$

There are 20 conditions for when a strategy can invade another strategy, and the calculations involve only standard algebraic manipulations, so we omit them. To simplify the expressions, we let $q_{\mathrm{s}} = b_{\mathrm{s}}/c$ and $q_{\mathrm{h}} = b_{\mathrm{h}}/c$, and we substitute a/c by a, so that the amount of aversiveness relates to the ratio q rather than b. The results are given in Table A.1, where a row mutant can invade the column strategy when the corresponding expression is positive.

For comparison to our original model, Table A.2 presents the same conditions, for $f_s =$

	**NA**	**BN**	**BA**	**AB**	**AA**
**NA**		$f_\mathrm{s}(1-rq_\mathrm{s})$ $+f_\mathrm{h}(1-r)q_\mathrm{h})$	$f_\mathrm{s}(1-rq_\mathrm{s})$ $+f_\mathrm{h}(1-rq_\mathrm{h}))$	$f_\mathrm{s}(1-r(q_\mathrm{s}-a))$ $+f_\mathrm{h}(1-r)(q_\mathrm{h}-a))$	$f_\mathrm{s}(1-r(q_\mathrm{s}-a))$ $+f_\mathrm{h}(1-r(q_\mathrm{h}-a))$
**BN**	$-f_\mathrm{s}(1-rq_\mathrm{s})$ $-f_\mathrm{h}(1-r))$		$-f_\mathrm{h}(q_\mathrm{h}-r))$	$f_\mathrm{s}ra$ $-f_\mathrm{h}(1-r))$	$f_\mathrm{s}ra$ $+f_\mathrm{h}(r-(q_\mathrm{h}-a))$
**BA**	$-f_\mathrm{s}(1-rq_\mathrm{s})$ $-f_\mathrm{h}(1-rq_\mathrm{h}))$	$f_\mathrm{h}(q_\mathrm{h}-r))$		$f_\mathrm{s}ra$ $-f_\mathrm{h}(1-q_\mathrm{h}+(1-r)a))$	$f_\mathrm{s}ra$ $+f_\mathrm{h}ra$
**AB**	$-f_\mathrm{s}(1-r(q_\mathrm{s}-a))$ $-f_\mathrm{h}(1-r))$	$-f_\mathrm{s}ra$ $+f_\mathrm{h}(1-r)q_\mathrm{h})$	$-f_\mathrm{s}ra$ $-f_\mathrm{h}r(1-q_\mathrm{h}))$		$f_\mathrm{h}(r-(q_\mathrm{h}-a))$
**AA**	$-f_\mathrm{s}(1-r(q_\mathrm{s}-a))$ $-f_\mathrm{h}(1-r(q_\mathrm{h}-a)))$	$-f_\mathrm{s}ra$ $+f_\mathrm{h}(q_\mathrm{h}-r(1+a)))$	$-f_\mathrm{s}ra$ $-f_\mathrm{h}ra)$	$-f_\mathrm{h}(r-(q_\mathrm{h}-a)))$	

Table A.1: Row mutant can invade single resident column strategy when the expression is positive.

	**NA**	**BN**	**BA**	**AB**	**AA**
**NA**		$-q_s+3q_h+4$	$-q_s-q_h+8$	$-q_s+3q_h-2a+4$	$-q_s-q_h+2a+8$
**BN**	q_s-7		$-4q_h+1$	$a-3$	$-4q_h+5a+1$
**BA**	q_s+q_h-8	$4q_h-1$		$4q_h-2a-4$	$2a$
**AB**	q_s-a-7	$3q_h-a$	$-q_h-a+1$		$-4q_h+4a+1$
**AA**	q_s+q_h-2a-8	$4q_h-2a-1$	$-2a$	$4q_h-4a-1$	

Table A.2: Row mutant can invade single resident column strategy when the expression is positive, when $f_s=f_h=r=0.25$.

$f_h = r = 0.25$ (all multiplied by 16 to avoid fractions). Figure A.2 presents the invasive conditions for the same parameter space as that used in the main paper, over the intervals $b_s \in [4, 8]$ and $b_h \in [0, 4]$. The coloured areas represent where the column strategy can be invaded by at least one of the other strategies, which means that the strategy is an ESS in the white areas.

The Deceptive nonhelper is an ESS in the same parameter space as in our original model. In line with the fact that the potential ESS area of the Deceptive helper was not altered by the introduction of aversive treatment in the original model, the Deceptive Helper has been mostly replaced by the Deceptive benign helper. The reason that the ESS territory of the Deceptive benign helper expands somewhat with increasing a, at the same time as the Opportunistic aversive helper increases (at a higher pace), is that it becomes mutually more difficult for the two strategies to invade each other with a larger a, as can be read from Table A.2.

The Honest benign helper occupies some of the area under which Honest Helper was an ESS without aversive treatment in the original model. However, it is a small subset of that of the Opportunistic aversive helper, which otherwise invades the Honest benign helper.

The Deceptive aversive helper is dominated by the Deceptive benign helper. The prevalence of aversive treatment thus depends on the Opportunistic aversive helper. The area under which it is an ESS increases with the aversiveness of the treatment (as it becomes more difficult for both the Deceptive benign helper and the nonhelper to invade), up to a threshold value (here $a = 3$), where there is no benefit to being opportunistic and providing aversive treatment is so non-beneficial to kin that the Honest benign helper invades, which is in turn invaded by the other strategies.

Importantly, the area under which the Opportunistic aversive helper is potentially an ESS covers that in which aversive treatment could introduce caregiving in the original model, and also some of the area where Nonhelper was previously the only ESS. In conclusion, the main differences to the dynamics of the original model are thus (1) there will be no aversive treatment where Deceptive Helpers constituted an ESS, but (2) the parameter space in which caregiving becomes possible due to aversive treatment expands.

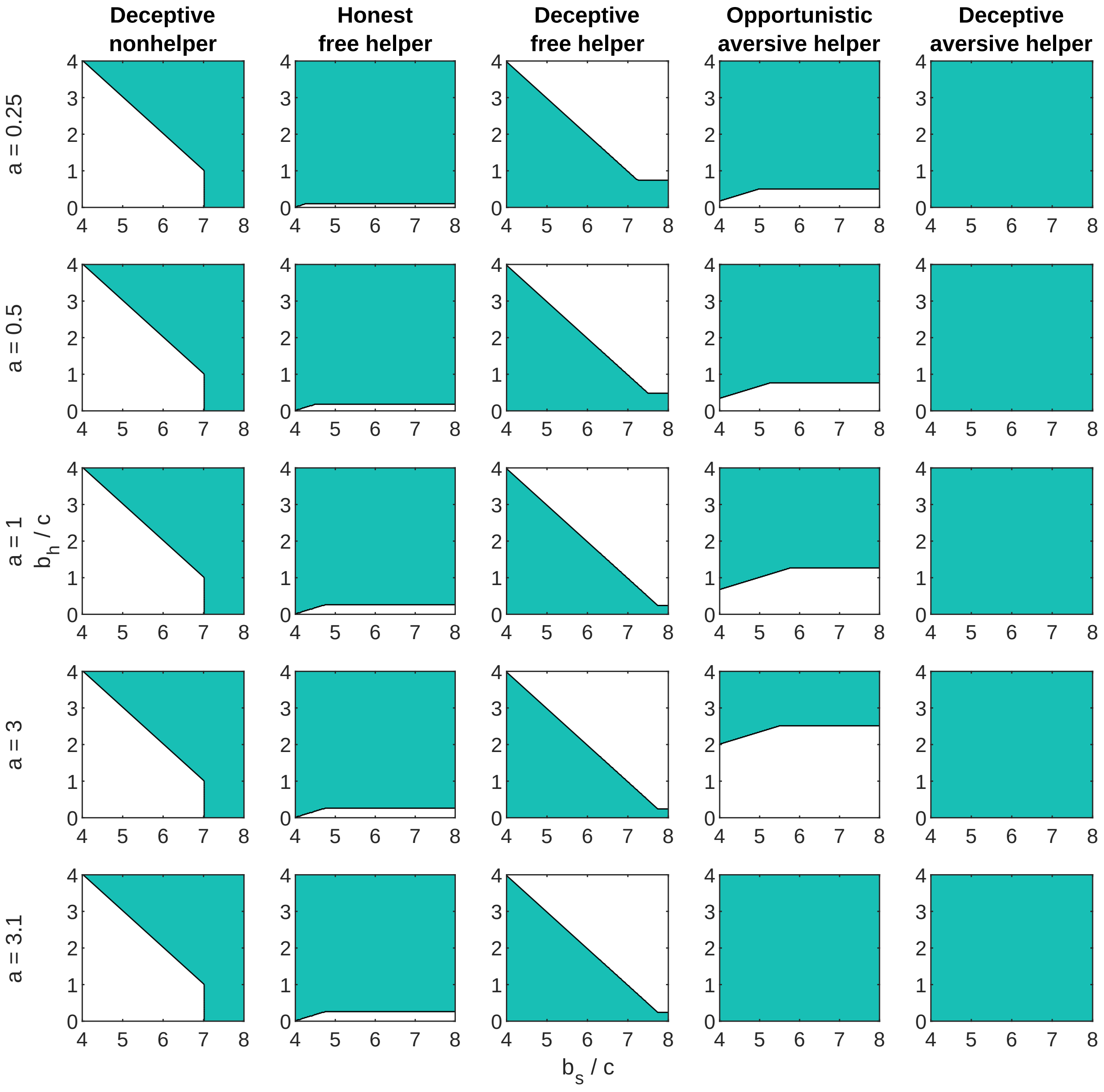

Figure A.2: Conditions under which the column strategy can be invaded by some other strategy in the coloured areas, for different values of aversiveness, a. The variables f_s, f_h and r are set to 0.25.
